# Relationships between Atherosclerosis and Plasma Antioxidant Micronutrients or Red Blood Cell Polyunsaturated Fatty Acids in People Living with HIV

**DOI:** 10.3390/nu11061292

**Published:** 2019-06-07

**Authors:** Katherine J. P. Schwenger, Bianca M. Arendt, Marek Smieja, David W. L. Ma, Fiona Smaill, Johane P. Allard

**Affiliations:** 1 Department of Medicine, University of Toronto, Toronto, ON M5S 1A1, Canada; kschweng@uhnresearch.ca; 2 Toronto General Hospital, University Health Network, Toronto, ON M5G 2N2, Canada; bianca.arendt@gmail.com; 3 Department of Clinical Epidemiology and Biostatistics, Health Research Methods, McMaster University, Hamilton, ON L8S 4L8, Canada; smiejam@mcmaster.ca; 4 Department of Pathology and Molecular Medicine, McMaster University Faculty of Health Sciences, Hamilton, ON L8S 4L8, Canada; smaill@HHSC.CA; 5 Department of Human Health and Nutritional Sciences, University of Guelph, Guelph, ON N1G 2W1, Canada; davidma@uoguelph.ca

**Keywords:** atherosclerosis, HIV, oxidative stress, polyunsaturated fatty acids

## Abstract

Background: People living with human immunodeficiency virus infection and acquired immune deficiency syndrome (HIV/AIDS) (PLWH) are at an increased risk of cardiovascular disease. Diet-related factors may contribute. The aim of this pilot study was to determine, in PLWH, the relationship between atherosclerosis assessed by carotid intima-media thickness (CIMT) and (A) plasma antioxidant micronutrients and oxidative stress or (B) red blood cell polyunsaturated fatty acids (RBC PUFA), particularly long chain omega-3 polyunsaturated fatty acids (n-3 PUFA). Methods: (A) In a cross-sectional study, subjects had CIMT evaluated by high resolution carotid artery ultrasound. Plasma was collected for vitamin C, measured by spectrophotometry; and alpha- and gamma-tocopherol, retinol, and malondialdehyde—a marker of oxidative stress—using high pressure liquid chromatography and fluorescence spectrophotometry. (B) In a prospective cohort study, other subjects had RBC PUFA measured at baseline, using gas chromatography, and CIMT assessed at baseline and repeated after 2 years. Clinical data was also collected. Results: (A) 91 PLWH participated. Only alpha- and gamma-tocopherol levels were positively correlated with CIMT. In a multivariate analysis, age and systolic blood pressure were significantly associated with CIMT with gamma-tocopherol near significance (*p* = 0.053). (B) 69 PLWH participated. At baseline, docosahexaenoic acid (n-3 PUFA) and the ratio of docosahexaenoic acid to arachidonic acid (n-6 PUFA) were significantly and negatively correlated with CIMT. However, a multivariate analysis failed to detect a significant relationship either at baseline or 2 years after. Conclusion: In addition to age and systolic blood pressure, atherosclerosis assessed by CIMT might be associated with higher serum gamma-tocopherol levels. There was a non-significant association between CIMT and RBC n-3 PUFA or the ratio of n-3 to n-6 PUFA.

## 1. Introduction:

People living with human immunodeficiency virus infection and acquired immune deficiency syndrome (HIV/AIDS) (PLWH) have an increased risk for cardiovascular disease (CVD), particularly individuals with long-term use of antiretroviral therapy (ART) [1,2]. Additionally, the traditional risk factors for CVD, including insulin resistance, diabetes, dyslipidemia, hypertension, chronic inflammatory states, and central fat deposition, are increasingly seen in PLWH, especially those on ART [1,2,3]. When studied in the general population, these CVD risk factors have been associated with endothelial dysfunction [4,5,6]. Endothelial dysfunction is characterized by increased permeability, altered endothelium-mediated vasodilatation, increased vascular reactivity, platelet activation and enhanced thrombogenicity, leukocyte adhesion, and monocyte migration [7]. 

Endothelial dysfunction is a result of increased oxidative stress in the vascular wall and is an independent predictor of CVD risk. It is seen in the early stages of atherosclerosis [6]. Oxidative stress plays a crucial role in the formation of plaque, and in addition with inherent vascular inflammation, may play a role in the pathogenesis of atherosclerosis, a main cause of CVD [8,9,10]. Atherosclerosis is a disease characterized by the excessive buildup of plaque on the inner walls of the arteries [11]. PLWH have been shown to have oxidative stress due to both chronic infection and side effects of ART [12,13,14]. Oxidative stress can also be influenced by the activities of antioxidant enzymes or levels of antioxidant micronutrients. Among these micronutrients, alpha- and gamma-tocopherol (vitamin E) are the most potent liposoluble antioxidant [15]. However, there are reports that they may also be associated with cardiovascular disease [16]. Other micronutrients that have antioxidant effects are vitamin C and retinol [17]. Levels of these micronutrients can be influenced by diet or presence of high oxidative stress [18]. Considering that PLWH are at risk of developing oxidative stress and micronutrient deficiencies, including antioxidants [19], it is of interest to evaluate the relationship between these antioxidants, the presence of oxidative stress, assessed by malondialdehyde (MDA) thiobarbituric acid reactive substances (TBARS) levels and atherosclerotic disease, since this has been minimally studied in PLWH. 

Poor diet quality can also contribute to an increased risk of CVD, specifically atherosclerosis. The Western diet, which is typically consumed in North America, is high in saturated fat, simple sugars, and salt, and low in fiber, fish oils, and antioxidants [20]. Research has identified different dietary factors that can decrease CVD risk, such as long chain omega-3 polyunsaturated fatty acids (n-3 PUFA) from fish oil [21]. n-3 PUFA in the form of eicosapentaenoic (EPA) and docosahexanoic acid (DHA) have been shown to decrease the risk of CVD morbidity and mortality [22,23] in the general population. The association between n-3 PUFA and CVD risk in PLWH has been minimally studied. Fish oil has been shown to reduce triacylglycerol in this population [24,25,26,27]. In addition, it has been shown to induce anti-inflammatory effects by increasing the formation of anti-inflammatory leukotriene B5 [26] from activated neturophils [26]. To our knowlegde, the association between n-3 PUFA and atherosclerosis has not been examined in PWLH.

PLWH are a unique population, not only because of their chronic infection, but also because of the drugs they take to suppress viral replication and to control metabolic abnormalities [28,29]. Therefore, the purpose of this pilot study was to evaluate in PLWH: (A) the association between antioxidant micronutrients, oxidative stress (MDA), and carotid intima-media thickness (CIMT) as a marker of atherosclerosis; and (B) the association between red blood cell (RBC) n-3 PUFA and CIMT. 

## 2. Materials and Methods

### 2.1. Study Population

Study participants were enrolled as part of the Canadian HIV-Vascular Study, which was a 5-year multi-center cohort study that has been previously published [30]. The population consisted of adults diagnosed with HIV, aged 35 years or older, presenting for routine clinical visits at one of five participating HIV clinics. They were enrolled regardless of cardiovascular risk factors or past cardiovascular disease and were followed yearly. For the cross-sectional study (A), a subset of patients consented to provide additional blood samples for the assessment of antioxidant micronutrients and oxidative stress marker (MDA) in addition to their baseline bloodwork and CIMT. For the prospective cohort study (B), another subset of patients consented to provide additional blood for the measurement of the RBC n-3 PUFA at baseline in addition to having their CIMT assessed at baseline and at 2 years. All subjects gave their informed consent for inclusion before they participated in the study. The study was conducted in accordance with the Declaration of Helsinki, and the protocol was approved by the Ethics Committee of University Health Network (08-0464).

### 2.2. Clinical and Demographic Data

At each clinic visit, a detailed questionnaire collected information on sex, age, smoking status, use and duration of HIV medication, nadir and current CD4 cell count, and viral load. Clinical measurements were taken and included blood pressure, height, weight, and waist circumference.

### 2.3. Laboratory Analyses

At baseline, fasting blood was taken to measure total-, high-density lipoprotein (HDL)-, and low-density lipoprotein (LDL)-cholesterol, triglycerides, glucose, and CD4-T-lymphocyte cell count. In addition, plasma was collected at baseline and red blood cells (RBC) were collected at every clinical visit in ethylenediaminetetraacetic acid (EDTA) containing tubes and stored at −80 °C until analysis. RBC was used to measure n-3 PUFA status, and plasma was used to assess antioxidant micronutrients and oxidative stress. 

For oxidative stress, MDA TBARS was analyzed by HPLC using a commercially available kit (Chromsystems Instruments and Chemicals GmbH, Munich, Germany). In addition, plasma antioxidant vitamins C, E, and retinol were measured. For vitamin C, plasma was stabilized with 100 g/L metaphosphoric acid (1:1) and analyzed by spectrophotometry [31]. Alpha- and gamma-tocopherol [32] (vitamin E) and retinol [33] were analyzed by HPLC and fluorescence spectrophotometry. 

For the PUFA study, total lipids were extracted from RBC and transmethylated [34]. Fatty acid methyl esters were measured in RBC using a gas chromatograph equipped with a flame ionization detector and a 100 mm × 0.25 mm SP2560 fused silica column [35]. The n-3 index was calculated as EPA + DHA (in % of total fatty acids) [36]. 

### 2.4. Carotid Intima-Media Thickness 

Subjects had their CIMT measured by high-resolution carotid artery ultrasounds. CIMT was assessed by calculating the mean of 12 CIMT measurements, located 1 and 2 cm above the carotid bifurcation, and 1 cm below, as previously described [11,37]. The images were videotaped and analyzed by computer-assisted software at the Hamilton General Site, Hamilton Health Sciences. This is a standardized technique, which has been validated in clinical trials, demonstrating high inter- and intra-observer reliability [37].

### 2.5. Statistics

Statistical calculations were performed by using SAS 9.3 software. Measured parameters were compared between groups using a paired *t*-test and Kruskal–Wallis test, followed by Wilcoxon ranked sum or chi-square and Fisher’s exact test, as necessary. For the multivariate analysis, CIMT was log (log_10_) transformed and all other data were presented as non-transformed, and multiple regression was used. Results were considered statistically significant at a *p*-value of less than 0.05. 

## 3. Results

### 3.1. Study A: Cross-Sectional Study Assessing the Relationship between Antioxidant Micronutrients, Oxidative Stress, and CIMT

The demographic and clinical data of the 91 PLWH participating in the study are shown in Table 1. Of the participants, 83.5% were male with a mean age of 47.8 years and a mean CIMT score of 0.09 (±0.03) cm (subclinical atherosclerosis defined as CIMT ≥ 0.9 mm [38]). Antioxidant micronutrient levels are reported in Table 1, where we also provide normal ranges found in healthy humans from our previous studies. Overall, PLWH had overall normal vitamin C concentrations, slightly lower retinol concentration, and higher alpha- and gamma-tocopherol concentrations.

Correlations between CIMT score and plasma antioxidant micronutrients or MDA were performed (Table 2). As a marker of oxidative stress, MDA did not correlate with CIMT (*p* = 0.587). As antioxidants, both gamma- and alpha-tocopherol were significantly positively correlated with CIMT measurements, whereas the other antioxidants were not. 

We then assessed subjects’ clinical characteristics according to alpha- and gamma-tocopherol tertiles (Table 3). Patients in the top third of alpha-tocopherol concentration were more likely to have a higher CIMT measurement (*p* = 0.022), higher ART use (*p* = 0.009), higher CD4 cell count (*p* = 0.046), and increased presence of plaque (*p* = 0.008). Patients with the top third of gamma-tocopherol concentrations followed a similar pattern, though this did not reach statistical significance. 

In a multivariate model of CIMT, clinical variables were adjusted using linear regression and included age, sex, systolic blood pressure, and the use of ART and lipid lowering medication. We found that age and systolic blood pressure were significantly associated with CIMT, while gamma-tocopherol was close to significance (Table 4). 

### 3.2. Study B: Prospective Cohort Study Assessing the Relationship between RBC n-3 PUFA and CIMT 

The demographic and clinical data of the 69 PLWH with PUFA measurements are shown in Table 5. Of the participants, 83.3% were male with a mean age of 49.1 years and a mean CIMT score of 0.08 (± 0.02) cm (subclinical atherosclerosis defined as CIMT ≧ 0.9 mm [38]). 

Overall, based on values from healthy controls in previous studies, PLWH had generally lower mean % linoleic, alpha-linolenic, eicosapentaenoic, and total n-3 acids, but higher docosahexaenoic and arachidonic acids [40,41]. Initially, correlations between CIMT score and baseline RBC PUFA were performed (Table 6). Only DHA and DHA/arachidonic acid (AA) ratio were significantly and negatively correlated with CIMT, whereas correlations with other PUFA were not significant. 

We then looked at tertiles of DHA, as well as the association of DHA with CIMT, using a multivariate analysis, but found no statistically significant association (data not shown). We also investigated if the significant increase in CIMT measurement (*p* < 0.0001) between baseline (0.08 cm (± 0.02)) and 2 years after baseline (0.09 cm (± 0.02)) was associated with baseline RBC PUFA, however, no statistically significant associations were found (data not shown). 

## 4. Discussion

Results suggest a potential association between high plasma gamma-tocopherol level and higher CIMT, but failed to detect any statistically significant independent relationships between CIMT and other antioxidant micronutrients, oxidative stress marker (MDA), or any RBC PUFA. In the prospective study, although there was an inverse relationship between DHA and CIMT at baseline, no association was found at baseline and 2 years after baseline when using multivariate analyses. 

Very few studies have investigated the association between serum antioxidant micronutrients, RBC PUFA, and CVD in PLWH. Micronutrient deficiencies are a concern in PLWH, as these deficiencies can cause accelerated disease progression and greater risk of treatment failure by impairing the immune system [19]. For example, vitamin C deficiency depresses the cell-mediated immune response and reduces the lymphocyte response, and vitamin E deficiency impairs lymphocyte proliferation and T-cell mediated function [42]. However, in our population sample, the antioxidant micronutrients were not within deficiency levels based on levels reported in our previous studies in healthy humans (29, 35) and our findings showed that high plasma levels of alpha- and gamma-tocopherol, not low levels, were associated with higher CIMT. Similar findings were reported in another cross-sectional study investigating alpha-tocopherol and other micronutrient concentrations in 298 PLWH [43]. The authors reported that the highest tertile of serum alpha-tocopherol concentration was associated with a higher CIMT score [43]. In addition, they found that no other micronutrients (serum selenium, zinc, and vitamin A) were associated with markers of atherosclerosis [43]. 

Studies investigating the use of vitamin E and risk of CVD in the general population are conflicting. On one hand, gamma-tocopherol increases the activity of nitric oxide synthase, which produces nitric oxide, known to relax vessels and improve cardiovascular health [44] with some observational studies showing that elevated vitamin E serum concentrations might be associated with a reduced risk of CVD [45,46]. On the other hand, other studies have shown that vitamin E supplementation provides no cardiovascular benefit, and in some cases may even causes harm [47]. A meta-analysis of randomized controlled trials found that high dosage vitamin E supplementation may increase all-cause mortality, specifically dosages greater than 150 IU/d [48]. One possible reason why vitamin E increases CVD risk could be its inhibiting effect on cytosolic glutathione *S*-transferases, which helps to detoxify drugs and endogenous toxins [49]. Therefore, the benefits of vitamin E are controversial and might differ depending on studied populations. Based on our results, PLWH may not benefit from vitamin E supplementation considering its association with elevated CIMT. However, there may be other confounders, such as drug effect, since those in the higher third of plasma alpha- and gamma-tocopherol were also higher users of ART, which can also be associated with atherosclerosis. Despite this, considering the body of literature on vitamin E and CVD, caution is required when considering vitamin E supplementation. 

Very few studies have investigated the role of n-3 PUFA in PLWH. n-3 PUFA, or fish oil, supplementation has been shown to be beneficial in general for people with hypertriglyceridemia [50] as well as PLWH [51,52]. In the present study, we found that DHA and the ratio of DHA/AA were negatively correlated with baseline CIMT score. High intake of n-3 PUFA has been associated with reduced CVD mortality in the general population [53,54] and has been found to inhibit pro-inflammatory pathways [55]. However, a randomized placebo-controlled study found that in HIV infected men on ART with moderate CVD risk, supplementation with n-3 PUFA did not improve endothelial function or endothelial activation (based on soluble vascular cell adhesion molecule-1 and soluble intercellular adhesion molecule-1). On the other hand, inflammation—specifically soluble tumor necrosis factor receptor 1—tended to improve [56]. A more recent randomized, double blind, parallel, controlled clinical trial also investigated the supplementation of n-3 PUFA and oxidative stress in PLWH, but no differences between the n-3 PUFA and the placebo group were reported [51]. However, the same study found that n-3 PUFA supplementation did significantly decrease triglycerides [51], which has also been found in other studies [27,57]. In a population-based study investigating the association of DHA and CIMT, it was found that DHA had a significant inverse association with CIMT [58], similar to our results at baseline. Additionally, a cross-sectional study investigated the adherence to a Mediterranean diet and the risk of subclinical atherosclerosis (defined as CIMT ≥ 0.9 mm or ≥1 carotid plaque) in PLWH and found that lower adherence to the Mediterranean diet was associated with subclinical atherosclerosis [59]. Though these results suggest that the Mediterranean diet, which is high in n-3 PUFA, is beneficial for protecting against atherosclerosis, they did not measure n-3 PUFA in the RBC. The ratios of n-3 to n-6 have been reported to affect the progression and regression of atherosclerosis [60]. One study found that DHA/AA ratio was negatively correlated with change in plaque volume in statin treated patients with coronary artery disease [61]. Furthermore, another study found that male patients with a low DHA/AA ratio had a higher risk of acute coronary syndrome compared with those with a high ratio [60]. Overall, most of the studies investigating PUFA in PLWH have focused on the effects of supplementation on triacylglycerol [24,25,26,27]. Our study is unique, as it measures the PUFA in the RBC, as well as CIMT as a marker for atherosclerosis, which also detects subclinical atherosclerosis. 

These studies have limitations—both are observational, therefore, associations are not proof of causality. Analyses were performed in a subset of patients participating in a larger cohort that required multiple measurements; this limited our sample size, as not all subjects agreed to participate in our studies. Evaluation of RBC PUFA at the 2-year follow-up was not possible because of the demands of the larger cohort study—RBC PUFA may have changed over time because of variations in diet. This may have affected CIMT at the 2-year follow-up and contributed to the lack of significant association between DHA and CIMT at the 2-year follow-up.

## 5. Conclusions

In conclusion, there is a potential positive association between CIMT and serum gamma-tocopherol in PLWH. Therefore, caution is required when considering vitamin E supplementation. Although an inverse relationship between DHA and CIMT was found at baseline, there was a non-significant association between CIMT and RBC n-3 PUFA when using multivariate analysis. Further studies should investigate longitudinally the role of antioxidant micronutrients and PUFA supplementation on CIMT and CVD. 

## Figures and Tables

**Table 1:** Demographic and clinical characteristics.

Variable	Value (*n* = 91)	Normal Range
Age (years)	47.8 (40.9, 53.1)	-
Sex (*n* (% males))	76 (83.5%)	-
BMI (kg/m^2^)	26.6 (22.7, 29.1)	-
Current smoker (*n* (%))	31 (34.1%)	-
Systolic BP (mm Hg)	126 (114, 137)	<120
Glucose (mmol/L)	5.56 (4.90, 5.80)	3.3–5.8
Cholesterol (mmol/L)	4.68 (3.83, 5.44)	<5.2
Triglycerides (mmol/L)	2.95 (1.24, 2.89)	0.45–1.71
LDL (mmol/L)	2.58 (1.99, 3.03)	<3.4
HDL (mmol/L)	1.15 (0.92, 1.27)	>0.91
ART use (n (%))	70 (76.9%)	-
CD4^+^ cell count (cells/uL)	504 (320, 700)	500–1500
CIMT (cm)	0.09 (0.07, 0.11)	Subclinical atherosclerosis defined as CIMT ≥ 0.9 mm [38]
Vitamin C (umol/L)	41.99 (25.94, 54.42)	11–114 [39]
Retinol (umol/L)	1.60 (1.26, 1.92)	1.80 ± 0.53 [32]
Gamma-tocopherol (umol/L)	6.11 (3.78, 7.42)	3.58 ± 1.73 [32]
Alpha-tocopherol (umol/L)	45.92 (30.79, 57.52)	21.96 ± 7.17 [32]
Malondialdehyde TBARS (uM)	0.82 (0.44, 1.02)	-

BMI, body mass index; BP, blood pressure; LDL, low-density lipoprotein; HDL, high-density lipoprotein; ART, antiretroviral therapy; CIMT, carotid intima-media thickness; TBARS, thiobarbituric acid reactive substances. Variables expressed as: mean (first quartile, third quartile) or *n* (percentage of patients) as appropriate.

**Table 2:** Correlation between CIMT and antioxidants.

Variable	Correlation Coefficient (*p*-Value)
Vitamin C (umol/L)	−0.030 (0.768)
Retinol (umol/L)	0.082 (0.428)
Gamma-tocopherol (umol/L)	0.251 (0.017)
Alpha-tocopherol (umol/L)	0.323 (0.001)
Malondialdehyde TBARS (uM)	0.0571 (0.587)

**Table 3:** Clinical characteristics by tertiles of alpha-tocopherol and gamma-tocopherol.

	Bottom Third (*n* = 31)	Middle Third (*n* = 29)	Top Third (*n* = 31)	*p*-Value
Alpha-tocopherol (umol/L)	<34.66	34.66–53.66	>53.66	
Serum alpha-tocopherol concentration (umol/L)	26.43 (22.54, 30.60)	43.65 (37.97, 48.67)	66.44 (57.43, 73.57)	
CIMT (cm)	0.08 (0.07, 0.10)	0.09 (0.07, 0.11)	0.10 (0.08, 0.12)	0.022
ART use (*n* (%))	22 (64.71%)	23 (71.88%)	31 (93.94%)	0.009
CD4^+^ cell count (cells/uL)	421 (180, 570)	516 (340, 665)	582 (405, 745)	0.046
Presence of plaque (*n* (%))	9 (26.47%)	16 (50%)	21 (63.64%)	0.008
Gamma-tocopherol (umol/L)	<4.16	4.16–6.24	>6.24	
Serum gamma-tocopherol concentration (umol/L)	3.17 (2.72, 3.85)	5.08 (4.74, 5.31)	10.01 (7.25, 12.18)	
CIMT (cm)	0.09 (0.07, 0.10)	0.09 (0.07, 0.11)	0.10 (0.08, 0.12)	0.139
ART use (*n* (%))	25 (80.6%)	18 (62.1%)	27 (87.1%)	0.074
CD4^+^ cell count (cells/uL)	504 (320, 680)	515 (360, 710)	710 (494, 280)	0.886
Presence of plaque (*n* (%))	10 (32.3%)	14 (48.3%)	19 (61.3%)	0.0733

CIMT, carotid intima-media thickness; ART, antiretroviral therapy. Variables expressed as: mean (first quartile, third quartile) or *n* (percentage of patients) as appropriate.

**Table 4:** Multivariate model of CIMT.

Parameter	Estimate	Standard Error	*p*-Value
Age (years)	0.015	0.003	<0.0001
Sex (female)	−0.078	0.061	0.202
Systolic blood pressure (mm Hg)	0.003	0.001	0.024
ART medication use	0.051	0.055	0.361
Lipid lowering medication use	0.103	0.061	0.097
Gamma-tocopherol	0.011	0.006	0.053

CIMT, carotid intima-media thickness; ART, antiretroviral therapy.

**Table 5:** Demographic and clinical characteristics of polyunsaturated fatty acids (PUFA) cohort.

Variable	Value (*n* = 69)	Normal Range
Age (years)	49.1 (43.3, 53.1)	-
Sex (*n* (% males))	55 (83.3%)	-
BMI (kg/m^2^)	27.0 (22.8, 29.6)	-
Current smoker (*n* (%))	21 (31.8%)	-
Systolic BP (mm Hg)	123 (112, 133)	<120
Glucose (mmol/L)	5.48 (5.00, 5.90)	3.3–5.8
Cholesterol (mmol/L)	4.83 (4.01, 5.45)	<5.2
Triglycerides (mmol/L)	2.15 (1.24, 2.14)	0.45–1.71
LDL-cholesterol (mmol/L)	2.75 (2.10, 3.38)	<3.4
HDL-cholesterol (mmol/L)	1.23 (0.99, 1.33)	>0.91
ART use (*n* (%))	58 (84.1%)	-
CD4^+^ cell count (cells/uL)	522 (345, 725)	500–1500
CIMT (cm)	0.08 (0.07, 0.10)	Subclinical atherosclerosis defined as CIMT ≧ 0.9 mm [38]
Linoleic acid (% total lipid)	12.43 (10.95, 13.72)	24.2 ± 3.61 [40]
Alpha-linolenic acid (% total lipid)	0.32 (0.21, 0.36)	0.37 ± 0.15 [40]
Arachidonic acid (% total lipid)	14.30 (13.41, 15.43)	10.6 ± 1.75 [40]
Eicosapentaenoic acid (% total lipid)	0.75 (0.51, 0.85)	1.84 ± 0.53 [40]
Docosahexaenoic acid (% total lipid)	3.87 (3.10, 4.46)	2.38 ± 0.78 [40]
Omega 3 (% total lipid)	4.63 (3.55, 5.28)	5.24 ± 1.32 [40]
Ratio of docosahexaenoic acid to arachidonic acid	0.28 (0.20, 0.34)	-
Ratio of eicosapentaenoic acid to arachidonic acid	0.05 (0.03, 0.06)	-

BMI, body mass index; BP, blood pressure; LDL, low-density lipoprotein; HDL, high-density lipoprotein; ART, antiretroviral therapy; CIMT, carotid intima-media thickness. Variables expressed as: mean (first quartile, third quartile), mean ± standard deviation, or *n* (percentage of patients) as appropriate.

**Table 6:** Correlation between CIMT and PUFA.

Variable	Correlation Coefficient (*p*-Value)
Linolenic acid (% total lipid)	−0.229 (0.059)
Alpha-linolenic acid (% total lipid)	0.038 (0.758)
Arachidonic acid (% total lipid)	0.079 (0.517)
Eicosapentaenoic acid (% total lipid)	−0.042 (0.731)
Docosahexaenoic acid (% total lipid)	−0.243 (0.044)
Omega 3 (% total lipid)	−0.209 (0.084)
Ratio of docosahexaenoic acid to arachidonic acid	−0.246 (0.042)
Ratio of eicosapentaenoic acid to arachidonic acid	−0.054 (0.658)
